# The Swedish National Pediatric Cataract Register (PECARE): Coexisting systemic disorders 2007–2023

**DOI:** 10.1111/aos.70054

**Published:** 2025-12-12

**Authors:** David Wackerberg, Jenny Gyllén, Birgitte Haargaard, Alf Nyström, Arzu Karatepe Hashas, Anna Linnarsson Wiklund, Eric Trocmé, Ulrika Kjellström, Kristina Tornqvist, Gunilla Magnusson

**Affiliations:** ^1^ Department of Ophthalmology, Västra Götalandsregionen Sahlgrenska University Hospital Mölndal Sweden; ^2^ Department of Clinical Neuroscience, Institute of Neuroscience and Physiology, Sahlgrenska Academy University of Gothenburg Gothenburg Sweden; ^3^ Private Ophthalmologist Copenhagen Denmark; ^4^ Department of Paediatric Ophthalmology and Strabismus St Erik Eye Hospital Stockholm Sweden; ^5^ Division of Ophthalmology and Vision, Department of Clinical Neuroscience Karolinska Institute, St Erik Eye Hospital Stockholm Sweden; ^6^ Department of Clinical Sciences, Ophthalmology, Skåne University Hospital Lund University Lund Sweden

**Keywords:** cataract surgery, comorbidity, congenital cataract, epidemiology, paediatric cataract, quality register, systemic disorders

## Abstract

**Purpose:**

To analyse the frequency and type of coexisting systemic disorders in children operated on for cataract in Sweden.

**Methods:**

Data were retrieved from the Swedish National Pediatric Cataract Register (PECARE) for children operated between January 1, 2007, and December 31, 2023 (*n* = 975), including follow‐ups at age 1, 2, 5 and 10. Cataracts due to uveitis, trauma or radiation, and lens extraction due to luxation were excluded. Genetic screening was not mandatory during this period.

**Results:**

Of the 872 children who remained after exclusions, 466 (53.4%) had unilateral cataracts and 406 (46.6%) had bilateral cataracts. Coexisting systemic disorders were found in 132/872 (15.1%), of which 5/132 (3.8%) were strongly suspected, 64/132 (48.5%) defined and 63/132 (47.7%) undefined. Overall, 20/872 (2.3%) were developmentally delayed without any systemic disorder diagnosis. Systemic disorder was present in 20/466 (4.3%) with unilateral cataracts, 112/406 (27.6%) with bilateral cataracts, 22/138 (15.9%) with bilateral inherited cataracts and 11/32 (34.4%) with parental consanguinity.

**Conclusion:**

Coexisting systemic disorders were present regardless of laterality, but more common among children with bilateral cataracts. Prevalence was similar among children with consanguineous parents, and lower among children with hereditary cataracts. National consensus regarding genetic screening for systemic disorders is needed.

## 1 BACKGROUND

Paediatric cataract is one of the most common causes of treatable blindness in children worldwide. In Sweden, the prevalence of children operated for cataract is 28/100 000 (Magnusson et al., 2018). The disease can present as a standalone eye disease, as the first symptom of a systemic disorder, or as part of a syndrome. The systemic disorders related to paediatric cataract are a heterogenic group representing metabolic disorders, chromosomal disorders, neurometabolic diseases, renal diseases, skeletal diseases, craniofacial syndromes, muscular diseases and dermatological diseases, among others (Bell et al., 2020; Lambert, 2023). Other known causes of paediatric cataract are inheritance, trauma, uveitis, radiation, corticosteroid treatment or malformation of the eye (Haargaard et al., 2004; Yi et al., 2011).

Genetic screening for systemic disorders has been suggested, with Gillespie et al. (2014) arguing that comprehensive screening will ‘lead to improved diagnostic and management outcomes through a stratified medicine approach’. The traditional evaluation strategy, suggested by sources such as the textbook *Taylor & Hoyt's Pediatric Ophthalmology and Strabismus* (Lambert, 2023) and the American Academy of Ophthalmology (EyeWiki, 2025), is to perform an evaluation regarding systemic disorders only when the suspicion arises. However, the superiority of genetic screening using next‐generation sequencing compared to the traditional approach has been demonstrated several times (Gillespie et al., 2016; Musleh et al., 2016). There are no guidelines or consensus regarding how children with cataract should be genetically or otherwise evaluated in Sweden, apart from the national natal screening for 26 severe and treatable congenital diseases (Table 1).

**TABLE 1:** Protocol of Swedish natal screening for 26 severe and treatable congenital diseases.

List of diseases included in the national natal screening program	ICD‐11
*Endocrine diseases*	
Congenital hypothyroidism*	5A00.0Z
Congenital adrenal hyperplasia	5A71.01
*Fatty acid oxidation disorders (beta‐oxidation defects)*	
Medium‐chain acyl‐CoA dehydrogenase deficiency	5C52.01
Long‐chain 3‐hydroxyacyl‐CoA dehydrogenase deficiency and 2 other specific defects in the trifunctional protein	5C52.01
Very Long‐chain Acyl‐CoA Dehydrogenase deficiency*	5C52.01
*Carnitine system disorders*	
Carnitine Palmitoyltransferase I deficiency	5C52.00
Carnitine‐acylcarnitine translocase deficiency	5C52.00
Carnitine palmitoyltransferase II deficiency*	5C52.00
Primary carnitine deficiency	5C52.00
*Organic acidurias*	
Isovaleric acidemia*	5C50.E0
Propionic acidemia*	5C50.E0
Methylmalonic acidemia*	5C50.E0
Glutaric acidemia type 1*	5C50.E1
Multiple acyl‐CoA dehydrogenase deficiency	5C52.01
Beta‐ketothiolase deficiency	5C50.DY
*Urea cycle defects*	
Citrullinemia	5C50.A3
Argininosuccinate lyase deficiency	5C50.A0
Arginase deficiency	5C50.A2
*Other amino acid metabolism disorders*	
Phenylketonuria*	5C50.0Z
Maple syrup urine disease	5C50.D0
Tyrosinaemia type 1	5C50.11
Homocystinuria*	5C50.E0
*Other inherited metabolic disorders*	
Biotinidase deficiency	5C50.E0
Galactosaemia*	5C51.4Z
*Other treatable genetic disorders*	
Severe combined immunodeficiency	4A01.10
Spinal muscular atrophy	8B61.Z

*Note*: Diseases marked with an * are linked to cataract development.

Coexisting systemic disorders have recently been investigated in two studies. Kessel et al. (2021) showed that the occurrence of associated systemic disease was 40.8% in bilateral childhood cataract in Denmark. In the United States, Nihalani and VanderVeen (2023) found syndromic, genetic or metabolic cataract in 23.8% of bilateral cases and 1.6% of unilateral cases, and hereditary cataract in 21.8% of bilateral cases and 0% of unilateral cases.

The Swedish Pediatric Cataract Register (PECARE) is part of the broader Swedish system of national quality registers, which aims to improve healthcare by continuously evaluating treatment outcomes and patient‐reported measures. National quality registers are funded by the Swedish Association of Local Authorities and Regions (SALAR) and are required to regularly report on quality improvements.

PECARE was established in 2007 as a sub‐register of the Swedish National Cataract Register. It collects comprehensive data on all children in Sweden undergoing lens extraction before the age of 8. The primary aim of PECARE is to improve paediatric cataract care and ensure equal treatment nationwide by continuously gathering and analysing data on demographics, preoperative factors (e.g. eye measurements, symptoms, referral causes, heredity and coexisting systemic disorders), surgical parameters and postoperative outcomes. Follow‐up data from examinations at 1, 2, 5 and 10 years of age are collected to monitor long‐term visual outcomes and complications. Despite the relatively low number of paediatric cataract cases annually, PECARE's high registration rate (85%–100% in recent years) enables meaningful national‐level analysis and quality assurance (Magnusson et al., 2023).

No mandatory genetic screening regarding coexisting diseases or syndromes has so far been provided by health care in Sweden, and it is our clinical experience that many children with cataract and coexisting developmental delay lack any clear explanation or diagnosis regarding the cause. The aim of this study was therefore to analyse the frequency and type of coexisting systemic disorders in children operated for paediatric cataract in Sweden.

## 2 METHODS

All children who underwent lens extraction registered between January 1, 2007 and December 31, 2023 were initially included (*n* = 975). Children with cataract due to uveitis, trauma or radiation and those operated for lens luxation were excluded (*n* = 103), resulting in the inclusion of 872 children for analysis.

Coexisting systemic disorder was defined as a condition with the potential to affect multiple body systems. The cataract was defined as a hereditary cataract when there was a family history of paediatric cataract in one or more biologically related family members. However, genetic testing was not conducted routinely for all patients. The presence of coexisting systemic disorders, developmental delay, consanguinity, heredity and persistent foetal vasculature (PFV) was analysed. Coexisting systemic disorders were categorised as follows: no coexisting disorder, intrauterine infection, metabolic disease, trisomy 13/18/21, other coexisting systemic disorders and unknown. Children from all groups except the first and last of these (no coexisting disorder and unknown) were analysed regarding the type of coexisting systemic condition. If a child was not classified as having a coexisting systemic disorder at the time of surgery but did in fact have a coexisting systemic condition registered at a follow‐up visit, they were reclassified accordingly. Patients with suspected coexisting systemic disorder were identified by analysing the free‐text comments for each patient in the web‐based PECARE form. The few cases (5/875, 0.06%) with information in the free‐text space regarding strong suspicion of coexisting systemic disorder were defined as having an undiagnosed coexisting systemic disorder.

The study was performed in accordance with the tenets of the Declaration of Helsinki as well as the General Data Protection Regulation (GDPR) and was approved by the Swedish Ethical Review Authority (reference numbers 2023‐03520‐01, and 2023‐07821‐02).

## 3 RESULTS

A total of 872 children met the inclusion criteria, comprising 466/872 (53.4%) with unilateral cataract and 406/872 (46.6%) with bilateral cataract (Table 2). The proportion of boys was slightly higher than the proportion of girls, both in the whole group (459/872, 52.6%) and in the laterality subgroups (unilateral: 237/466, 50.9%; bilateral: 222/406, 54.7%). Surgery before 1 year of age was performed in 390 children, 357 of whom had a registered follow‐up at age 1. The corresponding numbers for surgery prior to 2, 5 and 10 years of age were 466, 702 and 872 children respectively, and follow‐ups at 2, 5 and 10 years of age were registered in 393, 477 and 338 children, respectively. The total number of follow‐up visits registered was 1565.

**TABLE 2:** Demographics of the study population: *N* = 872 children, 466 with unilateral cataract and 406 with bilateral cataract.

Laterality of cataract	Unilateral (*n*)	Bilateral (*n*)	Total (*n*)
Male	237	222	459
Female	229	184	410
Coexisting systemic disorder	20	112	132
Developmental delay	11	73	84
Hereditary cataract	9	138	147
Consanguinity among parents	5	32	37
PFV	178	81	259
Operated <1 year of age	191	199	390
Follow‐up at 1 year of age	176	181	357
Operated <2 years of age	238	228	466
Follow‐up at 2 years of age	204	189	393
Operated <5 years of age	364	338	702
Follow‐up at 5 years of age	241	236	477
Operated ≤10 years of age	466	406	872
Follow‐up at 10 years of age	184	153	338

Abbreviation: PFV, persistent foetal vasculature.

### 3.1 Coexisting systemic disorder

The total number of children with defined or strongly suspected coexisting systemic disorders was 132/872 (15.1%). Of these, 5/132 (3.8%) were strongly suspected, 66/132 (48.5%) were defined as having a coexisting systemic disorder, and the remaining 63/132 (47.7%) lacked a definition regarding the type of coexisting systemic disorder. The largest subgroup was other coexisting systemic disorders (*n* = 87) followed by trisomy 13, 18 or 21 (*n* = 26) (Table 3).

**TABLE 3:** Defined and suspected coexisting disorders among children (*n* = 872) with unilateral (*n* = 466) and bilateral (*n* = 406) cataracts in Sweden, 2007–2023, classified according to ICD‐11.

ICD‐11 code	ICD‐11 description	Explanation	Unilateral	Bilateral
Intrauterin infection				2
*Metabolic disease*				12
5A61.5	Central diabetes insipidus	Wolfram syndrome		1
5C50.E0	Classical organic aciduria	Homocystinuria		1
5C51.4Z	Disorders of galactose metabolism, unspecified	Galactosaemia		1
*Trisomy*			3	23
LD40.0	Complete trisomy 21		3	16
*Other coexisting systemic disorder*			17	70
4A41.01	Juvenile dermatomyositis		1	
5A00.Z	Hypothyroidism, unspecified		1	
5A41	Hypoglycaemia without associated diabetes			1
5A61.0	Hypopituitarism	CHARGE syndrome		2
5C52.10	Disorders of cholesterol synthesis	Smith–Lemli–Opitz syndrome		1
5C53.Y	Other specified inborn errors of energy metabolism	Mitochondrial disease		1
5C60.0	Oculocerebrorenal syndrome	Lowe syndrome		3
8B00.Z	Intracerebral haemorrhage, site unspecified			1
8C70.6	Congenital muscular dystrophy	Muscle‐eye‐brain disease	1	
8C70.6	Congenital muscular dystrophy	Walker–Warburg syndrome		4
8D64.Z	Hydrocephalus, unspecified			3
BD1Z	Heart failure, unspecified		1	1
LA02.00	Myelomeningocele with hydrocephalus		1	
LA05.0	Microcephaly			2
LA05.Y	Other specified cerebral structural developmental anomalies			1
LA4Z	Clefts of lip, alveolus or palate, unspecified		1	
LA75.Y	Other specified structural developmental anomalies of lungs	Primary ciliary dyskinesia	1	
LB16.1	Hirschsprung disease		1	
LB30.1	Renal dysplasia		1	
LD24.D	Slender bone dysplasias	Seckel syndrome		1
LD24.GY	Other specified syndromic craniosynostoses	Saethre–Chotzen syndrome		1
LD27.0Y	Other specified ectodermal dysplasia syndromes	Gorlin–Goltz syndrome	1	
LD2C	Overgrowth syndromes	Beckwith–Wiedemann syndrome		1
LD2F.1Y	Other specified syndromes with multiple structural anomalies, not of environmental origin	Nance‐Horan syndrome		2
LD2F.1Y	Other specified syndromes with multiple structural anomalies, not of environmental origin	Stickler syndrome		1
LD41.00	Duplications of the long arm of chromosome 1	1q21.1 duplication	1	
LD44.G1	Deletions of the short arm of chromosome 16	16p11.2 deletion		1
LD7Z	Chromosomal anomalies, excluding gene mutations, unspecified			1
*Suspected coexisting systemic disorder*				5
N/A	N/A	Genetic test performed without diagnostic result		2
N/A	N/A	Suspected galactose kinase deficiency		1
N/A	N/A	Suspected spinal muscle atrophy		1
N/A	N/A	Suspected stunted growth		1

When analysing the unilateral cataract cohort, coexisting systemic disorder was found in 20/466 (4.3%) of the children, of whom 5/20 (25.0%) had developmental delay and 1/20 (5.0%) had hereditary cataract. Among children with bilateral cataract, coexisting systemic disorder was found in 112/406 (27.6%) of all children, 59/73 (80.8%) of children with developmental delay and 22/138 (15.9%) of children with hereditary cataract (Table 4).

**TABLE 4:** Overview of the study population and the interrelationships of the parameters analysed (*n* = 872 children).

	Coexisting systemic disorder	Consanguinity among parents	Developmental delay	Hereditary cataract	PFV
**Unilateral cataracts (*n* = 466)**	***n* = 20**	***n* = 5**	***n* = 11**	***n* = 9**	***n* = 178**
Coexisting systemic disorder	–	2	5	1	5
Consanguinity among parents	2	–	2	0	1
Developmental delay	5	2	–	0	5
Hereditary cataract	1	0	0	–	3
PFV	5	1	5	3	–
Male (*n* = 237)	15	2	5	4	91
Female (*n* = 229)	5	3	6	5	87
**Bilateral cataracts (*n* = 406)**	***n* = 112**	***n* = 32**	***n* = 73**	***n* = 138**	***n* = 81**
Coexisting systemic disorder	–	11	59	22	21
Consanguinity among parents	11	–	8	14	7
Developmental delay	59	8	–	13	18
Hereditary cataract	22	14	13	–	20
PFV	21	7	18	20	–
Male (*n* = 222)	69	20	46	68	38
Female (*n* = 184)	43	12	27	70	43

Abbreviation: PFV, persistent foetal vasculature.

A gender difference was seen regarding the prevalence of coexisting systemic disorders in both the unilateral group (15 boys and 5 girls, ratio 3:1) and the bilateral group (69 boys and 43 girls, ratio 1.6:1).

### 3.2 Developmental delay

Developmental delay was registered in 84/872 (9.6%) of the total group, 11/466 (2.4%) of the children with unilateral cataract, and 73/408 (18.0%) of the children with bilateral cataract. Overall, 20/84 (23.8%) of these children were not diagnosed with any coexisting systemic disorder.

### 3.3 Hereditary cataract

Hereditary cataract was present in 147/872 (16.9%) of the total group, 9/466 (1.9%) of the children with unilateral cataract, and 138/406 (34.0%) of the children with bilateral cataract. Hereditary cataract combined with coexisting systemic disorder was present in 1/9 (11.1%) of the children with unilateral hereditary cataract and 22/138 (15.9%) of those with bilateral hereditary cataract. Consanguinity was present in 14/138 (10.1%) with hereditary bilateral cataract.

### 3.4 Persistent foetal vasculature

PFV was present in 259/872 (29.7%) children, of whom 178/466 (38.2%) had unilateral cataract and 81/406 (20.0%) had bilateral cataract. PFV combined with coexisting systemic disorder was present in 5/466 (1.1%) children with unilateral cataract and 21/406 (5.2%) of children with bilateral cataract. In the subgroup with hereditary cataract, PFV was present in 3/466 (0.6%) of the unilateral cases and 20/406 (4.9%) of the bilateral cases.

### 3.5 Consanguinity

Consanguinity among parents was found in 37/872 (4.2%) of the total group, 5/466 (0.1%) of those with unilateral cataract, and 32/406 (7.9%) of those with bilateral cataract. In the unilateral cataract group, 2/5 children (40.0%) with consanguineous parents had a coexisting systemic disorder. The corresponding number in the group with bilateral cataract was 11/32 (34.4%).

## 4 DISCUSSION

The literature is sparse regarding the description of coexisting systemic disorders among children with unilateral cataract. This probably stems from the traditional assumption that unilateral cataract is primarily associated with ocular malformations, most commonly PFV. PFV was found in 29.7% of children with unilateral cataract in the present study, compared to 46.5% in the IoLunder2 study (Solebo et al., 2016) and 19.5% in a study by Nihalani and VanderVeen (2023). Furthermore, Nihalani and VanderVeen reported that 5/319 (1.6%) cases of unilateral cataract had syndromic, genetic or metabolic aetiology, compared to 20/466 (4.3%) children in the present study. Among those 20 individuals, diagnoses such as trisomy 13 (3 cases), Gorlin–Goltz syndrome (1 case) and muscle–eye–brain disease (1 case) were found. Nihalani and VanderVeen reported no cases of hereditary unilateral cataract, while the present study identified 9 children reported as such in PECARE.

The findings of hereditary unilateral cataract and unilateral cataract combined with systemic disorders might be explained by presuming that they represent asymmetric development of a future bilateral cataract. However, speaking against this argument is the fact that careful instructions are given when registering laterality in the PECARE form, and the definitions of both unilateral and bilateral cataract strictly represent the laterality of the cataracts as the condition before surgery. Furthermore, the patients in the present study all had several follow‐up registrations confirming the laterality of the cataract.

A further explanation regarding our findings connecting inheritance and unilateral cataract could be that PFV, which is the most common ocular malformation in unilateral cataract, can be inherited both as a dominant and as a recessive trait (Shastry, 2009). PFV was found in 3 of 9 cases with unilateral hereditary cataract in the present study, in contrast to the IoLunder2 study (Solebo et al., 2016) where no such cases were found. None of the children with hereditary cataract and PFV had any coexisting systemic disorders, but it is unclear whether the family member with cataract had PFV as well. Moreover, this still does not explain the unilateral presentation.

The fact that unilateral cataract in children can be associated with coexisting systemic disorders highlights the need for both the ophthalmologist and paediatrician to be aware that unilateral cataract can also be a sign of systemic disease and that further systemic evaluation might be necessary even in unilateral cases.

The prevalence of coexisting systemic disorder among children with bilateral cataract in the present Swedish study (27.6%) was lower than the prevalence reported from Denmark (40.8%) (Kessel et al., 2021) but slightly higher than findings from the United States (23.8%) (Nihalani & VanderVeen, 2023) and the UK (23.4%) (Solebo et al., 2016). The type of systemic disorder was only defined in approximately half of the children in the present study, leading to difficulties regarding the comparison of the different subgroups. Trisomy 21 was the largest subgroup in our study; this was reported in 16/406 (3.9%) bilateral cases, which is in line with the 7/211 (3.3%) bilateral cases in the abovementioned Danish study (Kessel et al., 2021). This similar incidence of trisomy 21 in both populations might lead to the suspicion that the difference in the prevalence of coexisting systemic disorders is due to more efficient detection of uncommon disorders in Denmark. This can be exemplified by the prevalence of metabolic disease, which was much higher in the Danish cohort (5.2%) than in our study (12/406, 3.0%) (Kessel et al., 2021).

Bilateral hereditary cataracts were slightly more common in the present study (33.9%) compared to the studies by Kessel et al. (2021) and Nihalani and VanderVeen (2023), which found a prevalence of 29.4% and 21.8%, respectively. However, the IoLunder2 study (Solebo et al., 2016) reported a higher frequency of hereditary cataract (43.3%). The reason for these differences remains unclear.

The combination of bilateral cataracts and PFV is portrayed as rare in the literature, with Nihalani and VanderVeen (2023) and Solebo et al. (2016) reporting it in 0% and 8.2% of patients respectively. In the present study, the frequency was more than twice as high, at 20.0%. However, the IoLunder2 study presented a strikingly high incidence of coexisting systemic disorders among children with bilateral cataract and PFV (7/12, 58%), compared to the present study (18.8%). The higher incidence of PFV in our study might originate from the surgeon's assessment of whether or not to operate on a cataract with coexisting PFV.

There is a well‐known relationship between consanguinity and both childhood malformations and rare genetic diseases (Hamamy et al., 2011; Temaj et al., 2022), but the literature regarding consanguinity and childhood cataract is sparse. Gillespie et al. reported that 10/36 (27.8%) of children with bilateral congenital cataract were of consanguineous parentage (Gillespie et al., 2014), which is substantially higher than the overall prevalence of bilateral cataract with consanguineous parentage in the present study (32/406, 7.9%). While our study presented a lower percentage of children born from consanguineous couples than Gillespie et al., the percentage was much higher than the estimated rate in Sweden (<1.0%) reported by Temaj et al. (2022). Our study showed that among children with consanguineous parents and bilateral cataract, the rate of coexisting systemic disorders was slightly higher than in the whole group of bilateral cataracts (34.4% vs. 27.6%).

A broad definition of coexisting systemic disorder was used to highlight the wide array of systemic disorders found among children with cataracts. Some of the syndromes found are well known to be associated with cataracts, while others, such as heart failure, exemplify the whole spectrum of coexisting systemic disorders present in children with unilateral or bilateral cataracts.

Comparing the results of the present study with the literature, it becomes obvious that if Sweden had a more standardised approach to genetic screening, several children with undetected coexisting systemic disorders could have been diagnosed. Consequently, the right healthcare resources could have been put in place. Allocating resources to optimise help for these children would benefit both the children and the health care system. When it comes to cases of isolated hereditary cataract, a genetic evaluation is also necessary, as parents usually want to know the cause of their child's diseases. Family planning is another important reason for performing a genetic test.

The strength of this study is the high coverage of the entire paediatric cataract population in Sweden over a period of 16 years. One of the main findings is also a limitation regarding the present purpose, namely that there are many children in the register who lack a specification regarding the type of coexisting systemic disorder. This lack of specification may be because the final diagnosis has not yet been made and might also be related to the number of children with developmental delay without any diagnosis of systemic disorder (*n* = 20). Finally, there may be children who were operated on for cataract in more recent years who have not yet received a diagnosis, either because they have not yet developed symptoms or because the diagnosis is not yet clear.

## 5 CONCLUSION

Coexisting systemic disorders are present in the paediatric cataract population regardless of laterality, although they are more commonly present, at least in one third, among children with bilateral cataracts. The prevalence of coexisting systemic disorders is similar among children with consanguineous parents, and lower among children with hereditary cataracts. There is an urgent need for national guidelines and consensus among paediatric ophthalmologists and paediatricians regarding evaluation and genetic testing of children with cataracts. The presence of hereditary cataracts and coexisting systemic disorders among children with unilateral cataracts highlights that these children should also be included in such guidelines.
